# Neuronal response variability as a product of divisive normalization; neurobiological implications at a macroscale level

**DOI:** 10.12688/hrbopenres.13062.1

**Published:** 2020-06-04

**Authors:** Kathy L. Ruddy, David M. Cole, Colin Simon, Marc T. Bächinger

**Affiliations:** 1 Trinity College Institute of Neuroscience and School of Psychology, Trinity College Dublin, Dublin, Dublin 2, Ireland; 2 Interdisciplinary Program in Neuroscience,, Utah State University, Logan, Utah, USA; 3 Neural Control of Movement Lab, Department of Health Sciences and Technology, ETH Zürich, Zurich, Switzerland

**Keywords:** Spiking, neuronal variability, neuronal oscillations

## Abstract

The occurrence of neuronal spikes recorded directly from sensory cortex is highly irregular within and between presentations of an invariant stimulus. The traditional solution has been to average responses across many trials. However, with this approach, response variability is downplayed as noise, so it is assumed that statistically controlling it will reveal the brain’s true response to a stimulus. A mounting body of evidence suggests that this approach is inadequate. For example, experiments show that response variability itself varies as a function of stimulus dimensions like contrast and state dimensions like attention. In other words, response variability has structure, is therefore potentially informative and should be incorporated into models which try to explain neural encoding. In this article we provide commentary on a recently published study by Coen-Cagli and Solomon that incorporates spike variability in a quantitative model, by explaining it as a function of divisive normalization. We consider the potential role of neural oscillations in this process as a potential bridge between the current microscale findings and response variability at the mesoscale/macroscale level.

The neural coding problem asks how richly graded stimuli are translated into action potentials. A notorious complication is that single spike trains do not follow a predictable schedule. Despite the stereotyped shape of spikes, their occurrence is highly irregular within and between stimulus presentations ( Kostal *et al*., 2007). The traditional solution has been to average responses across many trials. However with this approach, response variability is downplayed as noise, so it is assumed that statistically controlling it will reveal the brain’s true response to a stimulus ( Kostal *et al*., 2007). Accordingly, suspicions have long been harbored that averaging is an incomplete answer. For example, experiments show that response variability itself varies as a function of stimulus dimensions like contrast and state dimensions like attention ( Cohen & Maunsell, 2009; Hénaff *et al*., 2019; Mitchell *et al*., 2009; Rabinowitz *et al*., 2015; Ruff & Cohen, 2016). In other words, response variability has structure, is therefore potentially informative and should be incorporated into models which try to explain neural encoding. Coen-Cagli & Solomon (2019) take a unique approach to both quantitatively describe spike variability, and relate it to a specific mechanism – *divisive normalization*.

 In its basic formulation, divisive normalization refers to dividing the driving stimulus input by the pooled drive of an ensemble of inputs (i.e. the normalization signal). In a seminal writing, Heeger (1991) showed that divisive normalization can increase the dynamic range of primary visual cortex (V1) neurons to contrast and thereby improve contrast sensitivity under varying light conditions. Additionally, since it has been observed in multiple brain areas and circuits across species, it has been put forth as a canonical neural computation (i.e. similar to exponentiation and linear filtering ( Carandini & Heeger, 2011).

However, in the standard model of divisive normalization, the driving stimulus input and the normalization signal are deterministic variables ( Coen-Cagli & Solomon, 2019, Eq. 1.1). This simplification either ignores response variability or operates as if it has been averaged out. Coen-Cagli & Solomon (2019) integrate response variability into their model by replacing them with Gaussian distributions (Eq. 1.2). Then, because this idealized mathematical expression is computationally intractable, they generated approximate expressions for spiking mean and spiking variance as predicted by their model (Eq. 1.3–1.4). These approximations then allowed them to test their model’s predictions against real data. To do so they presented images of gratings of varying contrasts to anesthetized macaque monkeys while recording activity from V1 with microelectrodes. Thereby, they were able to verify that their model similarly predicted the effect of contrast on spiking mean.

The key finding of Coen-Cagli & Solomon (2019) was that normalization is inversely related to (a) spiking variability and (b) inter-neuronal correlation of variability. While offering a neurobiological mechanistic explanation of this effect was beyond the scope of their investigation, they cite inhibitory stabilization of network activity and specifically GABA _A_ inhibition as potential candidates providing a normalizing role. This has prompted us to consider the potential role of neural oscillations in this process, and particularly their dependence on inhibition, as a potential bridge between the current microscale findings and response variability at the mesoscale/macroscale level.

In considering how neural oscillations may be a key linking factor, it is essential first to understand the role of oscillation for constraining (and allowing) neuronal spiking to occur. Traditionally considered as an epiphenomenon or by-product of neuronal firing, it is now conversely believed that neuronal oscillations are more causal than consequential in driving neuronal activity (eg. Thut *et al*., 2012). Neurons are inherently sensitive to noise in the local brain environment, including fluctuations in temperature, pH, and nearby electrical potential changes. According to Buzsaki (2006), if the neuron’s membrane potential was fixed close to its firing threshold most of the time, then it may risk frequent accidental discharge due to small changes in excitation from the fluctuating background noise. On the contrary, if the membrane potential was fixed far below threshold, such that it would be unaffected by background noise, the energy requirement to depolarize the cell would be much too costly on the brain’s resources. Buzsaki (2006) suggests that the brain’s most energetically economical solution is to swing the membrane potentials of coordinated populations of neurons up and down, providing windows of increased and decreased likelihood of firing. This centrally coordinated threshold-adjusting mechanism is carried out by the interneuron system, by providing periodic bursts of inhibition, the duration of which determines the frequency of the oscillation. For example, if the inhibition is mediated by GABA _A_ receptors, the emergent oscillation is in the Gamma frequency (40–100 Hz).

Thus, oscillations constrain neuronal spiking into periodic windows of varying responsiveness, and hence may serve as a determinant of response variability. As in Coen-Cagli & Solomon (2019) this may manifest as unreliable spike responses to invariant external (sensory) stimulation. In this regard, a correspondence between the micro and macroscopic level can be made, as the same neuronal oscillations have been shown to systematically modulate characteristics of responses to repeated invariant sensory stimuli measured at the behavioral level ( Busch *et al*., 2009; Mathewson *et al*., 2009), and in muscle responses to exogenous transcranial magnetic stimulation ( Ruddy *et al*., 2018; Zarkowski *et al*., 2006).

For example, when measuring ongoing neural oscillations simultaneously during sensory or transcranial stimulation, the influence of the underlying oscillation on the variability of resulting responses can be demonstrated at multiple levels of the nervous system. In the motor system, when using TMS to stimulate primary motor cortex, motor evoked potentials (MEPs) in corresponding muscles are larger when the pulse arrives coinciding with low amplitude alpha (8–13 Hz) waves, or high amplitude gamma (30–80 Hz) waves recorded at the scalp ( Ruddy *et al*., 2018; Zarkowski *et al*., 2006) despite the intensity of the stimulation remaining fixed. Further, within the alpha rhythm recorded over sensorimotor cortex (often referred to in this context as the µ rhythm), it has been shown that MEPs are modulated systematically by the phase of the wave cycle; they are larger when stimulation occurs at the trough of the wave and smaller when at the peaks ( Schaworonkow *et al*., 2019; Zrenner *et al*., 2018). At a behavioral level, in the visual system when presented with the same (low contrast) stimulus on multiple trials, detection is higher when the underlying alpha oscillation in the visual cortex is at the trough of the wave cycle, rather than at the peak, and when the amplitude of the rhythm is generally low ( Busch *et al*., 2009; Mathewson *et al*., 2009). Similar results have been obtained in investigations of variability in visual evoked potentials ( Brandt & Jansen, 2009) and auditory evoked potentials ( Rahn & Basar, 2009).

Considering the aforementioned evidence, it is clear that background fluctuations in the brain’s electrical activity contribute substantially to the variation in responses recorded following repeated presentations of an invariant stimulus. Buzsaki (2006) claims that this apparently spontaneous brain-state variability is internally coordinated and may be the essence of cognition itself.

Coen-Cagli & Solomon (2019) provide a novel approach to account for modulations in neuronal response variability in a quantitative and descriptive fashion by demonstrating the role divisive normalization could play. It is unclear at present whether neural oscillations and the process represented mathematically by divisive normalization are mutually exclusive concepts. Modelling the additional influence of a dynamic oscillating normalization signal and reconciling this with the extant descriptions of interacting neurobiological and electrophysiological mechanisms is a challenge for the future of this field. Resolving this would ultimately contribute further towards bridging across different scales of measurement in neuroscience.

## Data availability

No data are associated with this article.
