# Supplementary material for: Neuronal response variability as a product of divisive normalization; neurobiological implications at a macroscale level
Source: HRB Open Res. 2020 Jun 4;3:34. [Version 1] doi: 10.12688/hrbopenres.13062.1 (PMC7687196; doi:10.12688/hrbopenres.13062.1)
Supplement: Peer review history

# Peer review: Reviewer response for version 1

**DOI:** 10.21956/hrbopenres.14158.r28246

**Published:** 2020-11-24

**Authors:** Valentine L Marcar

**Affiliations:** 1 Department of Psychology, University of Zürich, University Hospital Zürich, Zürich, Switzerland

The authors comment on the work published in the Journal of Neuroscience by Coen-Cagli and Solomon on their observation that as normalisation of the neuronal response increases, variability in discharge activity decreases. The paper by Rudd and co-authors offers neural oscillation as a plausible explanation for the observation of Coen-Cagli and Solomon. Rudd and colleagues use findings in the literature involving studies on human volunteers to back their explanation.

 While their arguments favouring neural oscillations and divisive normalisation as the underlying mechanism influencing response variability is clear, the link between neural oscillation and divisive normalisation is not. This issue would benefit from more extensive attention. It would also be helpful if the role of GABA _A_ or GABA _B_-receptors in regulating neural oscillations would be dealt with.

 The paper is well written, with arguments centring on divisive normalisation and neural oscillation clearly presented.

Are arguments sufficiently supported by evidence from the published literature or by new data and results?

Yes

Is the conclusion balanced and justified on the basis of the presented arguments?

Yes

Is the rationale for commenting on the previous publication clearly described?

Yes

Are any opinions stated well-argued, clear and cogent?

Yes

Reviewer Expertise:

Brain imaging using fMRI, NIRS and ERP. Link between stimulus property, neural response and the BOLD-signal and VEP.

I confirm that I have read this submission and believe that I have an appropriate level of expertise to confirm that it is of an acceptable scientific standard.

# Peer review: Reviewer response for version 1

**DOI:** 10.21956/hrbopenres.14158.r27503

**Published:** 2020-06-26

**Authors:** Lucia Li

**Affiliations:** 1 Department of Brain Sciences, National Institute for Health Research, Imperial College London, London, UK

This is a commentary on a recently published research article (in JoN) that provides model and macaque validation for the theory that divisive normalisation modulates response variability.

 The JoN paper finds that stronger normalisation of a neuron is associated with less variable response. The JoN paper does not posit the mechanism for this finding. The commentary here by Rudd *et al. *puts forward the theory that neural oscillations are a possible mechanism to explain this finding.

 The authors use findings from the non-invasive brain stimulation and sensory detection literature to expand on the theory.

 It is a well-written piece, which clearly and succinctly introduces two important concepts – divisive normalisation and neural oscillations. It also neatly summarises the paper on which it is commenting.

 As the authors point out, although both appear to be mechanisms underlying variability of neuronal response, it is not clear how neural oscillations and divisive normalisation links up. It would be helpful if the authors could expand on how the two might be linked e.g. is there evidence that strongly normalised neurons are those that have resting thresholds furthest away from the depolarisation threshold? Or is the link at a higher level, where high activity in GABA interneurons coincides with when neurones are most strongly normalised?  Even if there is not much hard evidence to link them, it would be helpful to know the ‘state of play’.

Are arguments sufficiently supported by evidence from the published literature or by new data and results?

Yes

Is the conclusion balanced and justified on the basis of the presented arguments?

Yes

Is the rationale for commenting on the previous publication clearly described?

Yes

Are any opinions stated well-argued, clear and cogent?

Yes

Reviewer Expertise:

Neuroscience, brain stimulation

I confirm that I have read this submission and believe that I have an appropriate level of expertise to confirm that it is of an acceptable scientific standard.
